# A Scoping Review of Contextual and Individual Factors for Hospital-Acquired Malnutrition Development in Adult Hospital Inpatients: Guiding a Proactive Preventative Approach

**DOI:** 10.3390/nu17182970

**Published:** 2025-09-16

**Authors:** Vivien Hui In Cheung, Ching Shan Wan

**Affiliations:** 1 Independent Researcher, Wollongong, NSW 2500, Australia; 2 Respiratory Research@Alfred, School of Translational Medicine, Monash University, Melbourne, VIC 3004, Australia; 3 National Health and Medical Research Council Centre of Research Excellence in Wiser Wound Care, Griffith University, Southport, QLD 4215, Australia

**Keywords:** hospital-acquired malnutrition, prevention, predictor

## Abstract

Background: Preventing nutritional decline during hospitalisation is imperative in reducing the development of complications such as malnutrition and pressure injuries. However, existing malnutrition screening and assessment tools employ a reactive rather than proactive approach, using predictors to identify inpatients who are already malnourished instead of those at risk of developing hospital-acquired malnutrition. Therefore, this review aimed to identify key contextual and individual factors contributing to nutritional deterioration and their interrelatedness, and to inform strategies for preventing hospital-acquired malnutrition. Methods: A scoping review of five databases (Medline, CINAHL, Embase, All EBM Reviews and PsycINFO) up to June 2024 was conducted to include English-language studies that reported statistically significant risk factors for changes in nutritional status during hospitalisation. A directed acyclic graphing method was used to visualise the interlinkage between contextual and individual risk factors identified. PRISMA Extension for Scoping Reviews was followed in reporting. Results: Of 8215 retrieved abstracts, 51 studies were included. Four contextual (ward type; food service satisfaction; medical-related mealtime interruption; nutrition care collaboration) and four individual factors (nutritional status prior admission; hospital length of stay; multimorbidity; disease acuity) were found to significantly predict nutritional decline during hospitalisation and were closely interrelated. Conclusions: More contextual risk factors are modifiable, suggesting a need for organisational strategies to optimise collaborative nutrition care and improve patient satisfaction with hospital food services to promote early nutritional intervention, particularly within the first three days of admission and for inpatients with multimorbidity, high disease acuity, or pre-existing malnourishment.

## 1. Introduction

Hospital-acquired malnutrition, also known as hospital malnutrition, is defined as any decline in nutritional status occurring during a hospital stay, regardless of whether patients are malnourished or not upon admission [1]. Among the two types of hospital malnutrition—starvation-related malnutrition and disease-related malnutrition—only 10% of disease-related malnutrition is considered non-preventable, arising from disease-associated inflammation despite adequate dietary intake [1]. Although hospital-acquired malnutrition is largely preventable, nutritional decline during hospitalisation is common. Notwithstanding the underestimated prevalence of hospital-acquired malnutrition worldwide [2], up to 65% of inpatients experience nutritional decline [3], and a pooled hospital-acquired malnutrition incidence of 25.9% is observed in acute and subacute settings [4], depending on diagnostic criteria, nutritional assessment tools and timeframes for assessment.

Additionally, hospital-acquired malnutrition remains a persistent patient safety issue in acute care, imposing significant financial burdens on healthcare systems due to prolonged hospital stay and avoidable readmission [4]. It also compromises patient quality of life, increasing the risk of having other hospital-acquired complications, such as pressure injuries and surgical site infections [4,5]. Given its highly preventable nature and association with other hospital-acquired complications, preventing hospital-acquired malnutrition is listed in safety and quality standards as an urgent governmental and organisational priority worldwide [6]. It highlights the importance of identifying inpatients at risk of nutritional decline and providing timely, appropriate nutritional support to prevent hospital-acquired malnutrition development, and subsequent complications [5].

Despite its impact, hospital-acquired malnutrition is often overlooked, affecting over one-fourth of inpatients [7]. In 2022, national and international clinical nutrition societies endorsed a position statement declaring nutrition care as a human right, emphasising the importance of optimising nutritional therapy to prevent and treat hospital malnutrition [8]. This also signifies the global concern over the unacceptably low access to nutrition care for people with chronic or acute diseases [8]. Timely and appropriate medical nutrition therapy is imperative to prevent and treat malnutrition, reduce unnecessary economic burden in resource-limited hospital settings and improve hospital patient safety [9,10].

Preventing hospital-acquired malnutrition is more cost-effective than treating it [11]. Identifying at-risk inpatients is a crucial first step for initiating timely medical nutrition treatments. However, existing malnutrition screening and assessment tools employ a reactive rather than proactive approach, focusing on identifying already malnourished inpatients, rather than inpatients who are at risk of developing hospital-acquired malnutrition [12]. Nutritional decline during hospitalisation is also often undetected because much research only measures malnutrition prevalence at a single time point, overlooking changes in nutritional status over time during the hospital stay [13].

A proactive approach requires understanding the factors that contribute to nutritional decline, including contextual and individual factors contributing to the hospital-acquired malnutrition development, and their interrelationships [3]. However, existing reviews have provided the pooled prevalence of hospital-acquired malnutrition and perceived causes of hospital-acquired malnutrition [3], leaving the evidence on significant contributing factors and their interrelatedness uncertain.

This review, therefore, aimed to identify key contextual and individual factors contributing to changes in nutritional status among hospitalised adults and to explore how these risk factors interrelate. The findings will provide important insights into developing a proactive approach to prevent hospital-acquired malnutrition and related complications.

## 2. Materials and Methods

A scoping review was systematically conducted following the PRISMA Extension for Scoping Reviews (PRISMA-ScR) [14] to explore the breadth and depth of existing evidence. This approach was chosen because the parameters for measuring changes in nutritional status and associated risk factors were too broad to allow for evidence synthesis in a traditional systematic review. To ensure replicability, the literature search and data analysis procedures are outlined below.

### 2.1. Search Strategy

A literature search was conducted for all articles indexed by Medline, CINAHL, Embase, All EBM Reviews and PsycINFO up to June 2024. The same search strategy was applied across all five databases, combining medical subject headings and keywords related to three search term concepts: “risk factors”, “malnutrition” and “hospital”. Medical subject headings and keywords were amended accordingly for each database. Search term concepts were combined using Boolean “AND”. Alternative spellings and synonyms were combined using Boolean “OR”. The full list of search teams using Embase is provided in Supplementary Table S1.

### 2.2. Eligibility Criteria and Study Selection

Eligible studies were English-language cohort studies, case–control studies, case series, or randomised controlled trials published in peer-reviewed journals that investigated statistically significant risk factors influencing changes in nutritional status among adult hospital inpatients, defined as changes in malnutrition status measured using validated screening or assessment tools or anthropometry. In line with the recent paradigm shift in hospital-acquired malnutrition research, only studies that measured nutritional status at least twice during hospitalisation were included [3].

The screening and selection process was managed in Covidence systematic review software (Veritas Health Innovation, Melbourne, Australia. Available at www.covidence.org. accessed on 1 June 2024). Title/abstract and full-text screening, study selection, and data extraction were conducted independently by two authors (C.S.W. and V.C.), with disagreements resolved through discussion.

### 2.3. Data Extraction

For each included study, the following information was extracted: first author, country, year of publication, study design, sample size, hospital settings, ward type, methods used to measure changes in nutritional status, follow-up duration, and factors examined to assess the association with changes in nutritional status. Quality assessment was not performed, as the aim of this scoping review was to map the breadth of available evidence rather than assess its quality [15]. No additional data were sought from the study authors.

### 2.4. Data Analysis

Data analysis was conducted by one researcher (C.S.W.) and findings were discussed with a second researcher (V.C.) to finalise results. NVivo Version 14 (Lumivero, Denver, CO, USA, 2023) was used to support narrative synthesis of significant factors associated with changes in nutritional status. To synthesise how contributing factors are interrelated, a directed acyclic graph method was used to illustrate the interlinkage between contextual and individual risk factors that significantly influence nutritional status during hospitalisation. By conceptualising mediators and theorising mechanisms that contribute to the development of hospital-acquired malnutrition, it provides a fundamental basis for identifying hospitalised patients at risk of developing malnutrition and determines which modifiable factors to be prioritised in prevention strategies. Such an approach allows us to design proactive interventions to prevent hospital-acquired malnutrition and subsequently other complications such as hospital-acquired pressure injuries.

## 3. Results

**Figure 1:**
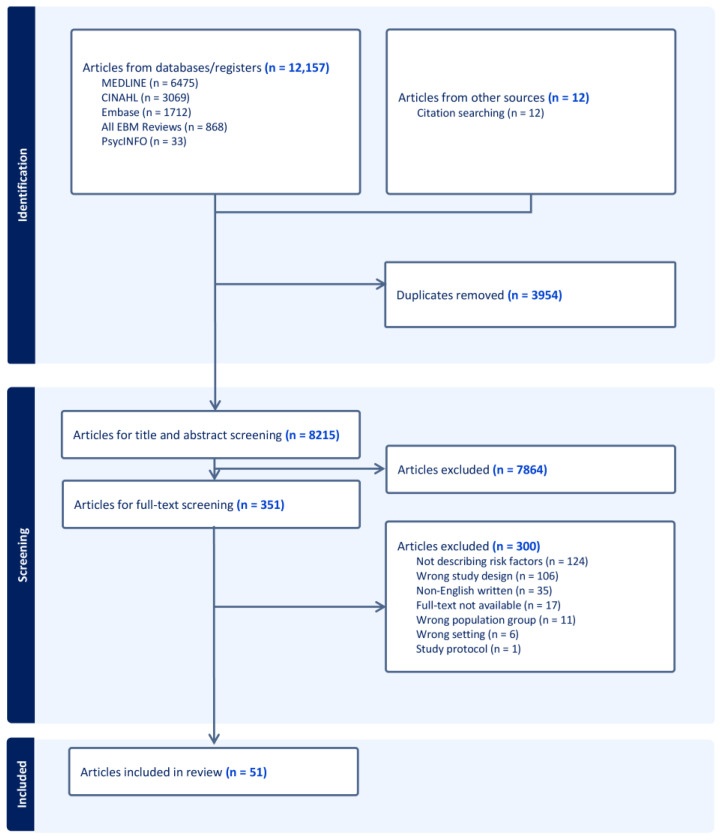
PRISMA diagram for article selection.

Figure 1 illustrates the systematic article selection process. After removing duplications, 8215 articles were initially screened based on title and abstract, followed by the retrieval of 351 articles for full-text screening. In total, 51 articles met the inclusion criteria for evidence synthesis. Most exclusions were due to wrong study design (n = 106; nutritional status only measured once), or analysis limitations (n = 124; examined risk factors associated with either nutritional status at admission or discharge, rather than changes in nutritional status during hospitalisation).

### 3.1. Main Characteristics of the Included Studies

Table 1 summarises the main characteristics of the included studies. Most were cohort studies (n = 47), conducted at a single hospital site (n = 40), used either validated nutritional screening or assessment tools to measure nutritional status (n = 32), and had nutritional status measured repeatedly at both hospital admission and discharge (n = 22). Most studies were conducted in Japan (n = 6), China (n = 6), Australia (n = 5) and Spain (n = 5). Data were collected from a diverse range of hospital ward settings.

**Figure 2:**
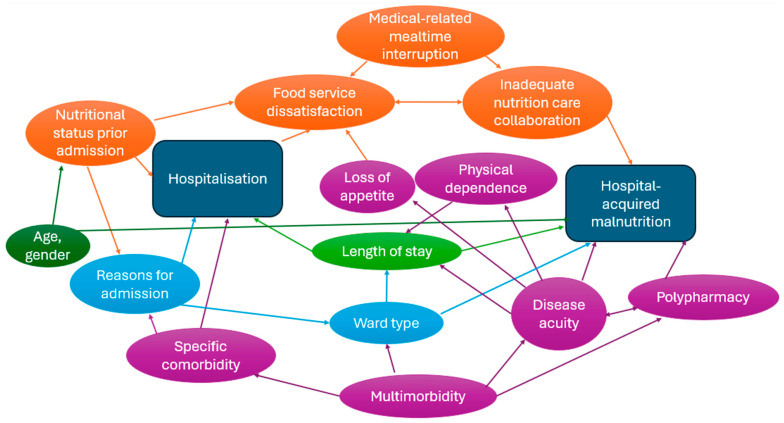
Directed acyclic graph illustrating the interrelatedness of factors contributing to hospital-acquired malnutrition. Rectangle boxes indicated the start and end points of the directed acyclic graph. Oval circles were factors contributing to hospital-acquired malnutrition. Orange colored circles were the identified nutrition-related contextual factors. Purple colored circles were the identified disease-related individual factors. Other factors were colored in different colors.

Following the synthesis of evidence on factors contributing to nutritional decline during hospitalisation, four contextual-level and four individual-level overarching factors were identified to be interrelated, as described below. The interrelatedness between factors identified is presented as a directed acyclic graph in Figure 2.

### 3.2. Contextual Factors

Four contextual factors were found to be significantly associated with hospital-acquired malnutrition: (1) ward type, (2) food service satisfaction, (3) medical-related mealtime interruption, and (4) nutrition care collaboration.

#### 3.2.1. Ward Type

Admission ward type, which is closely related to the reason for hospital admission, is a key contextual factor influencing the degree of nutritional deterioration during hospital stay. Compared to other ward types, patients admitted to sub-acute, geriatric, and rehabilitation wards are more likely to maintain or improve their nutritional status [3]. Although considerable heterogeneity exists in the rates of nutritional deterioration across various wards, primarily due to differences in the quality of routine hospital nutrition care provided across hospital sites, deterioration is more likely to be observed in surgical wards than in medical wards within the same hospital [18,22]. For example, the proportion of deteriorated patients in a Canadian hospital was slightly higher in the surgical ward (27.2%) compared to the medical ward (19.8%) [18]. Surgical procedure is one of the significant predictors of nutritional deterioration in hospitals, after adjusting for confounders [22]. Interestingly, among patients in medical wards who experienced nutritional deterioration, the presence of a surgical procedure and dissatisfaction with hospital food were the only two factors significantly associated with the decline [18].

#### 3.2.2. Food Service Satisfaction

Hospital meal quality is a key contributor to patients’ appetite [1,3,18,24,33,42,50,61]. The taste, appearance, and aroma of hospital foods were significantly associated with patients’ food intake rather than portion sizes, tiredness, breathing or swallowing difficulties [18]. At a tertiary hospital in Turkey, there was a continuous decline in oral intake from the fourth day of admission until discharge [50]. The primary reasons for reduced food intake during hospitalisation were patient refusal (65%) [24,53] and lack of appetite (56%) [33], highlighting the importance of optimising hospital food services as a motivator to increase patient oral intake and prevent hospital-acquired malnutrition.

#### 3.2.3. Medical-Related Mealtime Interruption

Medical-related mealtime interruptions refer to any interruption of enteral nutrition or oral intake due to medical procedures in hospitals, such as preoperative fasting or fasting for assessment procedures [24]. In an Australian hospital, preoperative fasting was the primary reason for enteral nutrition interruption, accounting for 33% of cases [24]. Similarly, in two Australian hospitals, fasting for surgery or procedures accounts for approximately 20% of oral intake interruptions [1,24]. Contrarily, in two German hospitals that implemented the NutritionDay initiative—which involves annual audit of hospital nutrition data to improve nutrition care practices and reduce malnutrition—only 3% of patients experienced oral mealtime interruptions due to medical examinations, surgery, or tests [33]. It implies that raising awareness and optimising collaborative hospital nutrition care has the potential to reduce medical-related mealtime interruptions, thereby preventing hospital-acquired complications, such as malnutrition and pressure injuries.

#### 3.2.4. Nutrition Care Collaboration

Preventing nutritional deterioration during hospitalisation requires fostering a collaborative work culture that emphasises inpatient nutrition care [23,36,57,64], as nutrition is often considered a low clinical priority among the multidisciplinary team, except for dietitians [3]. One study in Spain revealed that seven in ten oncology patients at risk of malnutrition at discharge had not received any nutritional support during hospitalisation [51], despite their increased risk of malnutrition due to disease-related cachexia. At an Australian hospital, the default hospital menu was insufficient to meet the nutritional needs for wound healing [38], highlighting the importance of additional input from dietitians in supplement prescription to ensure nutritional adequacy. At another Australian hospital, the inability to prescribe and provide an appropriate nutrition support from nurses and dietitians within the first week of hospitalisation was the only significant independent predictor of nutritional deterioration, after adjusting for frailty, delirium risk and age (odds ratio and 95% confidence interval: 2.3 (1.0, 5.1)) [13].

In some hospitals in China, Spain and Croatia, whether or not patients received nutritional care plan from dietitians was not associated with their risk of malnutrition [19,49,63]. This may be due to malnutrition risk assessment being inaccurately performed by nursing staff [58], or because dietitians might have only requested nurses to monitor inpatients’ dietary intake and weight without prescribing additional nutritional intervention [26], particularly for palliative care patients [60]. Nevertheless, effective and timely communication between nurses, dietitians and food services staff is critical in optimising nutrition care from malnutrition risk screening, appropriate nutritional care planning and nutrition support provision.

### 3.3. Individual Factors

Four individual-level factors were found to be significantly associated with hospital-acquired malnutrition: (1) nutritional status prior admission, (2) hospital length of stay, (3) multimorbidity, and (4) disease acuity.

#### 3.3.1. Nutritional Status Prior Admission

Patients who were already undernourished at admission, indicated as unintentional weight loss, low body mass index (BMI), low skinfold thickness, or low calf circumferences [18,19,28,30,33,55,56,59], were significantly more likely to experience nutritional deterioration during hospitalisation. Pre-existing malnutrition is considered as the primary individual risk factor for in-hospital nutritional deterioration [54]. In a small Japanese study including 26 surgical patients, those with a low preoperative BMI were twice as likely to experience nutritional deterioration, even after adjusting for confounders such as postoperative chemoradiotherapy [37]. In another small Chinese study of 44 patients undergoing liver transplantation, patients with preoperative nutritional risk had an 8.7-fold increased likelihood of postoperative nutritional deterioration compared to those without pre-existing nutritional risk [41].

Age and gender are common factors contributing to the association between pre-admission nutritional status and nutritional deterioration during hospitalisation. Patients aged 65 or above were at significantly higher nutritional risk at both admission and discharge compared to younger patients [19,30,45,51,55,56,59,63], due to more fluctuations in appetite among older adults [52]. However, the evidence on gender differences in pre-admission nutritional status and how gender influences hospital-acquired malnutrition rates remains inconsistent. While some studies report that women are at significantly higher risk of hospital-acquired nutritional decline than men [19,28,42], others suggest that men are more likely to experience nutrition deterioration during hospitalisation [18,54].

#### 3.3.2. Hospital Length of Stay

Hospital length of stay is a significant bi-directional contributing factor to nutritional deterioration, creating a self-perpetuating cycle: the longer the hospital stay, the greater the degree of nutritional decline, which in turn further prolongs hospital stay [16,20,21,32,53,54]. For instance, in a study among hospitalised cancer patients in Spain, more than 3% weight loss from baseline was observed in 45.3% of patients admitted for 11 to 20 days, and in 48.5% of those who admitted for more than 20 days [51]. Another Spanish study reported that patients who developed malnutrition during hospitalization had twice the length of stay compared with those who remained well-nourished at discharge [19]. In-hospital nutritional deterioration mostly occurred after a week of hospitalisation [13]. Interestingly, meeting more than 60% of protein requirements during the initial three days of hospitalisation has been shown to significantly shorten length of stays by an average of 4.4 days [62], highlighting the importance of early nutritional intervention in preventing nutritional decline and improving medical outcomes.

#### 3.3.3. Multimorbidity

Multimorbidity is another key individual-level predictor of nutritional deterioration during hospitalisation [19,20,22,33]. It is also closely interrelated with polypharmacy, another significant individual-level predictor [25]. Polymedicated patients had a 14.3% higher malnutrition rate at discharge compared to those who were non-polymedicated [19]. Several health conditions have been significantly associated with increased risk of nutritional deterioration during hospitalisation, including diabetes [19,33], cancer [18,19,37,49,56,63], cardiovascular disease [1,19,21,22,55,65], anorexia [48], depression [48,53], cognitive impairment [26], pressure injury [29], anaemia [22], and permanent catheter infections [22].

The number of long-term health conditions a patient has is also significantly associated with longer hospital stay and more weight loss, further contributing to nutrition deterioration [22]. Similarly, the total number of medications used at admission was independently associated with poorer nutritional status at discharge [43]. In particular, the use of more than five medications was significantly associated with nutritional deterioration after adjusting for confounders [35].

#### 3.3.4. Disease Acuity

Additionally, disease severity has a significant positive correlation with nutritional deterioration during hospitalisation [27,34,42,54,63]. Disease severity refer to the progression of particular health conditions such as malignancy and the need for haemodialysis, existence of complications, or increased dependency for physical functioning and feeding [22,29,30,46,54,63]. Complications that were found to be significantly associated with nutritional deterioration, due to their role in prolonging hospital stay, include fever [21], new infection diagnosis [18,33,65], physical or swallowing impairments after stroke [27,28,65], taste alterations after chemoradiotherapy [37,40], and gastrointestinal symptoms such as diarrhoea, constipation, oedema, and vomiting [22,30,40,47]. After adjusting for length of stay, poor oral health and impaired functional status were independently and significantly associated with nutritional decline [25,48], particularly among ventilator-dependent patients [29]. In relation to oral intake dependency, post-stroke patients with reduced levels of consciousness had a 2.8-fold increased risk of malnourishment compared to those without [65]. On the other hand, patients with pneumonia were twice as likely to experience nutrition decline compared to those without the condition [41,65]. Interventions such as swallowing training [39] and exercise training [17] were found to improve nutritional status during hospitalisation [31,44].

## 4. Discussion

The existing evidence on factors significantly associated with nutritional deterioration during hospitalisation sheds light on identifying approaches for preventing hospital-acquired malnutrition. Among the four contextual and four individual-level significant predictors identified, most contextual factors are modifiable, while most individual-level factors are not. It implies the significance of organisational-level nutrition initiatives in proactively preventing hospital-acquired malnutrition, and subsequently, improving associated patient safety indicators such as pressure injury prevention. NutritionDay is a global initiative to raise organisational awareness of the malnutrition issue in healthcare settings by conducting a one-day cross-sectional malnutrition audit [33]. It provides a unique opportunity for hospitals to monitor and benchmark the institutions’ nutrition care on an interventional level [33]. To align with NutritionDay’s goal of facilitating hospitals in proactively preventing hospital-acquired malnutrition, it would be worthwhile recommending participating hospitals to conduct repeated malnutrition audits one week later. This would help hospitals identify contextual factors contributing to nutritional decline in some inpatients, thereby guiding continuous improvement in nutrition care.

To prevent hospital-acquired malnutrition, it is of paramount importance to implement evidence-based, organisational-level strategies that promote an interdisciplinary nutrition culture. Such strategies have been shown to be cost-effective in improving health outcomes [66]. Emerging evidence from nationwide initiatives demonstrates that promoting a nutrition-focused work culture and reallocating roles among multidisciplinary teams can strengthen nutrition care collaboration and efficiency [12,67,68,69]. Our findings also highlight that patient food refusal is a primary contributor to nutritional deterioration during hospitalisation [24]. Addressing this requires targeted and evidence-based strategies, such as mealtime assistance programs (e.g., introducing volunteer support for patients in need). This should be adopted in place of Protected Mealtimes, an international initiative that needs to be disinvested, as it provides negligible benefits for hospitalised patients [70]. Hospital food quality has also become a new research focus of implementation science, aiming to identify areas for improvement and barriers to enhancing patient satisfaction with hospital food service [71,72]. Given the variability in the implementation of nutrition care initiatives across hospitals worldwide, conducting needs assessment through quality improvement audits is a critical first step to tailoring interventions in different hospital contexts. The overarching goals of such initiatives are to foster an interdisciplinary nutrition care culture, provide appropriate mealtime assistance, and improve food service satisfaction, ultimately reducing hospital-acquired malnutrition. As patient food refusal is a key contributor to nutritional deterioration, future interventions should be co-designed with patients to enhance engagement and involvement in hospital nutrition enhancement projects to tailor their needs.

Considering the priority-driven and resource-limited nature of hospital settings, it is practical to prioritise wards where patients are at greatest risk of hospital-acquired malnutrition. Our findings identified surgical procedure as a key predictor of nutritional deterioration, implying the significance of effectively implementing the Enhanced Recovery After Surgery (ERAS) protocol. ERAS is an evidence-based interdisciplinary perioperative clinical guideline designed to reduce postoperative complications and improve patient safety [73]. However, three key components of the ERAS guidelines—avoiding prolonged preoperative fasting, administering preoperative carbohydrate loading, and initiating early postoperative nutrition—are only partially or insufficiently implemented worldwide [74], despite being emphasised in ESPEN practical guidelines [75]. Traditional, non-evidence-based dietary restrictions persist in perioperative care due to multifaceted challenges in compliance with the ERAS guidelines [7]. In contrast, the successful implementation of ERAS guideline recommendations can foster early nutritional intervention, improve patient nutritional intake adequacy [62,76,77], thereby shortening hospital stays and preventing postoperative nutritional deterioration. Therefore, future efforts to support the uptake of ERAS guidelines are suggested to begin by co-designing an interdisciplinary perioperative dietetic care model with key stakeholders that supports the implementation of ERAS guidelines, can be adopted within the existing hospital infrastructure, and is sustainable. This co-developed perioperative dietetic care model will be evaluated in future trials for its feasibility, appropriateness and effectiveness.

Prolonged length of stay, high disease acuity, pre-existing malnutrition, and multimorbidity are identified as the four key individual-level factors contributing to nutritional deterioration during hospitalisation. As previously mentioned, prolonged length of stay can be prevented through implementing timely organisational-level nutritional interventions. Inadequate nutritional intake or insufficient nutritional support are predominant barriers to meeting nutritional requirements during episodes of acute illness that require hospitalisation [1,3]. Nearly 90% of hospital-acquired malnutrition is preventable, except for disease-related malnutrition caused by severe disease acuity, where nutritional needs exceed the patients’ metabolic capacity [1,3]. Therefore, identifying patients at risk of nutritional decline at admission is a crucial initial step in providing the right nutritional support to the right individuals at the right time. While the existing nutritional screening and assessment tools are well-validated for predicting malnutrition and associated adverse clinical outcomes, they are not designed to identify which patients can benefit from nutritional support the most [78]. Our findings shed light on the aspects of disease acuity that impair nutritional intake and the patient characteristics that predict nutritional deterioration during hospitalisation. Given the overlap between these individual-level risk factors and the key aspects of frailty (physical dependence, disease severity and multimorbidity), incorporating frailty assessment at admission may help target individuals benefiting from early nutritional interventions. Exploring the utility of frailty tools in malnutrition prevention appears to be an important future research direction.

In summary, this review provides focused insights into the synthesis of evidence on predictors of nutritional decline during hospitalisation and their interrelatedness (using the directed acyclic graphing method) from studies that measured changes in nutritional status during hospitalisation and quantitatively investigated relevant risk factors. However, the field is still in its infancy, as reflected by the inclusion of 51 studies, most with small sample sizes. To capture the breadth of evidence, we applied broader inclusion criteria for the study design, which introduced greater heterogeneity in the quality of evidence. Furthermore, substantial variation in the instruments used to measure changes in nutritional status and contributing factors precluded meta-analysis synthesis. Most included studies employed nutritional screening tools with high sensitivity, enabling early detection of malnutrition risk or malnourishment; however, this may potentially overestimate our findings when these tools were used to measure changes in nutritional status. Future studies would benefit from using anthropometric measures to assess changes in nutritional status more accurately.

## 5. Conclusions

Evidence highlights the need for initiatives that foster interdisciplinary collaboration in nutrition care, ensure appropriate mealtime assistance, and provide early nutritional support for surgical inpatients. Implementation science offers promise in increasing the uptake and sustainability of these evidence-based practices within routine hospital nutrition care, helping to prevent hospital-acquired malnutrition and associated complications, such as pressure injuries and surgical site infections. This may be further achieved by identifying inpatients at risk of nutritional decline during hospitalisation, based on factors such as multimorbidity, disease acuity, and pre-admission malnutrition, followed by providing early nutritional support within the first three days of hospitalisation. Additional research is required to guide the development of organisational-level interventions that improve patient satisfaction with hospital food services and ERAS guideline implementation. Future research is also needed to evaluate the effectiveness of frailty assessment tools in identifying patients at risk and providing early nutritional support to prevent nutritional decline during hospitalisation.

## Figures and Tables

**Table 1:** General characteristics of included studies.

Author, Year	Sample (n), Country	Study Design	Settings and Ward Types	Nutritional Status Measurement	Follow-Up Period	Risk Factors Examined
Abahuje, 2020 [16]	279, Africa	Cohort	Surgical ward in a hospital	SGA	Weekly	Age, gender, disease diagnosis, multimorbidities, disease acuity, and length of stay
Akazawa, 2022 [17]	200, Japan	Cohort	Subacute and rehabilitation wards in a hospital	Skinfold thickness	During hospital stay	Age, sex, disease diagnosis, and malnutrition risk at admission
Allard, 2015 [18]	424, Canada	Cohort	Medical and surgical wards at multiple hospital sites	SGA	≥7 days of hospitalisation	Age, sex, CCI, disease, disease acuity, appetite, and food service satisfaction
Alvarez Hernandez, 2012 [19]	1707, Spain	Cohort	General, orthopedic, geriatric and rehabilitation wards at multiple hospital sites	NRS-2002	During hospital stay	Age, BMI, polypharmacy and disease diagnosis
Antonelli Incalzi, 1996 [20]	302, Italy	Cohort	Medical and geriatric wards in a hospital	SNA	Weekly	Age, BMI, number of comorbid diseases, disease diagnosis and ADL score
Axelsson, 1988 [21]	100, Sweden	Cohort	Stroke unit in a hospital	Skinfold thickness	Weekly	Age, gender, disease diagnosis, disease acuity, and medical treatment
Borrego Utiel, 2011 [22]	77, Spain	Case–control	Haemodialysis clinic at a hospital	Body weight	≥4 days of hospitalisation	Age, gender, months on dialysis, CCI, disease acuity, length of stay, disease diagnosis, and medical treatment
Botero, 2024 [13]	130, Australia	Cohort	Medical and surgical wards in a hospital	SGA	Weekly	Age, CCI, Frailty Index, delirium risk, and nutrition care plan prescribed or not
Caccialanza, 2015 [23]	30, Italy	Case–control	Non-surgical wards in a hospital	Body weight	Not reported	Energy and protein intake, and nutritional supplementation
Chapple, 2016 [24]	47, Australia	Cohort	Neuro-trauma clinic in a hospital	Body weight	Not reported	Type of nutritional support, and interruptions to dietary intake
Chen, 2009 [25]	306, Taiwan	Cohort	Medical and surgical wards in a hospital	MNA	During hospital stay	Disease diagnosis, number of multimorbidities, polypharmacy, BI, length of stay, and surgical treatment
Cheng, 2019 [1]	23, Australia	Cohort	In a quaternary hospital	SGA	Not reported	Age, gender, CCI, and disease diagnosis
Collins, 2016 [26]	248, Australia	Cohort	Geriatric rehabilitation wards in a hospital	MNA	During hospital stay	Age, gender, functional independence, and disease diagnosis
Crary, 2013 [27]	67, United States	Cohort	Stroke clinic in a hospital	MNA	Hospital stay up to 7 days	Disease diagnosis and disease acuity
Diendere, 2018 [28]	222, Africa	Cohort	Stroke units in multiple hospitals	Skinfold thickness	Weekly	Age, gender, disease acuity, attempt to eat, and dietary support received
Flury, 2023 [29]	252, Switzerland	Cohort	Rehabilitation ward at a hospital	SNST	Not reported	Age, gender, BMI, type of nutritional support, feeding assistance required, and disease diagnosis
Fu, 2017 [30]	310, China	Cohort	Cancer ward in a hospital	NRS-2002	Not reported	Disease acuity, appetite, and treatment-related side effects
Gayo, 2014 [31]	76, Spain	Cohort	Special care clinic in a hospital	MNA	During hospital stay	Disease diagnosis, disease acuity and BI
Gobel, 2022 [32]	112, Turkey	Cohort	Intensive care units at a hospital	NRS-2002	21 days	Age, dietary intake, and type of nutritional support
Graeb, 2021 [33]	156, Germany	Cohort	Geriatric wards at 2 hospitals	MUST	During hospital stay	Age, gender, length of stay, BMI, reasons for admission, polypharmacy, multimorbidities, nutritional status prior to admission, dietary intake, reasons for reduced oral intake and nutritional support provided
Gubari, 2019 [34]	64, Iran	Cohort	Intensive care unit in a hospital	SGA	Weekly	Disease acuity
Hafsteinsdottir, 2010 [35]	196, Netherlands	Cohort	Neurological ward in a hospital	MNA	10 days	Gender, disease diagnosis, polypharmacy, walking deficits, BI
Incalzi, 1996 [36]	286, Italy	Cohort	Medical and geriatric wards in a hospital	Midarm circumference	During hospital stay	Dietitian support, appetite, and feedback on food service
Kagifuku, 2020 [37]	26, Japan	Cohort	Cancer ward in a hospital	BIA	During hospital stay	Age, gender, perioperative BMI, cancer treatment
Liang, 2008 [38]	31, Australia	Cohort	Vascular unit in a hospital	Body weight	Repeated at 5–6 days	Dietary intake and appetite
Lin, 2003 [39]	61, Taiwan	Case–control	Patients at eight hospitals	Midarm circumference	Weekly	Swallowing ability and swallowing training provided
Lin, 2020 [40]	465, China	Cohort	Cancer wards in a hospital	PG-SGA	Before and after chemotherapy	Treatment side effects, appetite, and disease acuity
Liu, 2024 [41]	44, China	Cohort	Liver transplant ward in a hospital	NRS-2002	Weekly	Age and frailty
Liu, 2016 [42]	170, China	Cohort	Haematological ward in a hospital	NRS-2002	Not reported	Unintentional weight loss and dietary intake changes after treatment
Matsumoto, 2022 [43]	257, Japan	Cohort	Rehabilitation ward in a hospital	GNRI	During hospital stay	Nutritional status at admission, disease diagnosis, rehabilitation therapy performed, functional dependence, dietary intake, CCI, length of stay, polypharmacy, and swallowing function
Mosselman, 2013 [44]	73, Netherlands	Cohort	Neurological ward in a hospital	MNA	10 days	Gender, discharge destination, disease type, swallowing abilities, and BI
Nematy, 2013 [45]	114, Iran	Cohort	Medical wards in a hospital	NRS-2002	7–10 days	Age, gender, disease diagnosis, and dietary intake
Padillo, 1999 [46]	39, Spain	Cohort	Medical ward in a hospital	Skinfold thickness	During hospital stay	Disease acuity and dietary intake
Paillaud, 2006 [47]	88, France	Cohort	Advanced cancer ward in a hospital	Skinfold thickness	Monthly	Dietary intake and physical functional status
Patel, 2008 [48]	100, United Kingdom	Cohort	Geriatric ward in a hospital	Body weight	Monthly	Disease acuity and reasons for inadequate dietary intake
Pavicic Zezelj, 2020 [49]	160, Croatia	Cohort	Medical wards in a hospital	NRS-2002	During hospital stay	Type of nutritional support and dietary intake
Pekmezci, 2018 [50]	47, Turkey	Cohort	Infectious disease wards in a hospital	NRS-2002	Weekly	Age, gender, dietary intake and length of hospital stay
Planas, 2016 [51]	401, Spain	Cross-sectional	Oncological ward at multiple hospitals	NRS-2002	During hospital stay	Age and BMI at admission
Pourhassan, 2021 [52]	191, Germany	Cohort	Geriatric wards in a hospital	MNA-SF	During hospital stay	Age, gender, disease acuity, CCI, appetite and dietary intake
Purnamasari, 2023 [53]	55, Indonesia	Cohort	Medical wards in a hospital	Body weight	During hospital stay	Reasons for fasting during hospitalisation and length of stay
Roganovic, 2022 [54]	650, Serbia	Cohort	Medical wards in a hospital	Skinfold thickness	During hospital stay	Gender, disease diagnosis, disease acuity, length of stay, nutritional status at admission and mobility worsening
Sato, 2019 [55]	205, Japan	Cohort	Acute wards in a hospital	GNRI	During hospital stay	Age, BI and disease diagnosis
Shim, 2013 [56]	435, Korea	Cohort	Cancer wards in a hospital	PG-SGA	Weekly	Age, gender, preoperative weight loss, cancer type and treatment type
Shimazu, 2021 [57]	426, Japan	Cohort	Rehabilitation ward in a hospital	BIA	During hospital stay	Age, gender, disease diagnosis, CCI, dietary intake, dietary prescription frequency, length of stay
Sidenvall, 1993 [58]	18, Sweden	Cohort	Geriatric ward in a hospital	Body weight	During hospital stay	Nutritional support from a multidisciplinary team
Sunaga, 2022 [59]	982, Japan	Cohort	Cardiac ward in multiple hospital sites	GNRI	During hospital stay	Age, gender, disease acuity, length of stay, frailty and disease diagnosis
Venzin, 2009 [60]	211, Switzerland	Cohort	Medical ward in a hospital	Body weight	During hospital stay	Treatment type, fasting for diagnostic reasons, disease diagnosis, disease acuity, and nutritional treatment plan
Wright, 2021 [61]	262, Indian	Cohort	Geriatric wards at five hospitals	MST	During hospital stay	Nutritional support received and nutritional status before admission
Yeung, 2017 [62]	115, Canada	Cohort	Colorectal wards at two hospitals	MST	During hospital stay	Dietary intake and ERAS protocol implemented or not
Yu, 2013 [63]	687, China	Cohort	Surgical wards at two hospitals	NRS-2002	Two weeks or until the time of discharge	Disease type and disease acuity
Zhang, 2015 [64]	760, China	Cohort	Stroke units at eight hospitals	BMI	Two weeks	Age, gender, disease diagnosis, type of nutritional support, disease acuity and dysphagia

ADL: Activities of Daily Living; BI: Barthel Index; BIA: Bioelectrical Impedance Analysis; BMI: Body mass index; CCI: Charlson Comorbidity Index; ERAS: Enhanced Recovery After Surgery; GNRI: Geriatric Nutritional Risk Index; MNA: Mini-Nutritional Assessment; MNA-SF: Mini Nutritional Assessment Short Form; MUST: Malnutrition Universal Screening Tool; NRS: Nutritional Risk Screening; PG-SGA: Patient-Generated Subjective Global Assessment; SGA: Subjective Global Assessment; SNA: Subjective Nutritional Assessment; SNST: Spinal Nutrition Screening Tool.

## Data Availability

No new data were created or analyzed in this study. Data sharing is not applicable to this article.

## Supplementary Materials

The following supporting information can be downloaded at: https://www.mdpi.com/article/10.3390/nu17182970/s1, Table S1: Full search strategy for Embase.
