# Supplementary material for: A Scoping Review of Contextual and Individual Factors for Hospital-Acquired Malnutrition Development in Adult Hospital Inpatients: Guiding a Proactive Preventative Approach
Source: Nutrients. 2025 Sep 16;17(18):2970. doi: 10.3390/nu17182970 (PMC12472483; doi:10.3390/nu17182970)
Supplement: Supplementary file 1 [file nutrients-17-02970-s001.zip › nutrients-3853446-supplementary.pdf]

Supplementary Table S1: Full search strategy for Embase

1	("Protein energy malnutrition" or "Protein-energy malnutrition" or "Protein calorie malnutrition" or "Protein-calorie malnutrition" or malnourish* or undernutrition or malnutrition or "disease-related malnutrition" or "disease related malnutrition").ti. or ("Protein energy malnutrition" or "Protein-energy malnutrition" or "Protein calorie malnutrition" or "Protein-calorie malnutrition" or malnourish* or undernutrition or malnutrition or "disease-related malnutrition" or "disease related malnutrition").ab.
2	protein calorie malnutrition/ or malnutrition/
3	1 or 2
4	(Hospital* or "Acute care" or inpatient).ti. or (Hospital* or "Acute care" or inpatient).ab.
5	hospital patient/ or hospital/ or aged hospital patient/
6	4 or 5
7	(predictor* or risk*).ti. or (predictor* or risk*).ab.
8	3 and 6 and 7
